# Supplementary material for: NANOG prion-like assembly mediates DNA bridging to facilitate chromatin reorganization and activation of pluripotency
Source: Nat Cell Biol. 2022 Apr 28;24(5):737–47. doi: 10.1038/s41556-022-00896-x (PMC9106587; doi:10.1038/s41556-022-00896-x)
Supplement: Supplementary file 1 — Supplementary Methods and Supplementary Fig. 1. [file 41556_2022_896_MOESM1_ESM.pdf]

nature portfolio

https://doi.org/10.1038/s41556-022-00896-x

Supplementary information

NANOG prion-like assembly mediates DNA bridging to facilitate chromatin reorganization and activation of pluripotency

In the format provided by the
authors and unedited

Supplementary Methods:

Construction of Mammalian Expression Plasmids. Constructs used in the study are listed in Supplementary Table 3. DNA and primer sequences used in the study are listed in Supplementary Table 4. All generated constructs and mutations were confirmed by DNA sequencing (Eurofins Genomics).

pcDNA-H6f-Nanog. The Nanog gene was amplified from the pEP4 E02S EN2L (Addgene #20922) using the primer sets (44-1F/44-1R) and subcloned into XbaI/KpnI-digested pcDNA3.1 (-) vector (Invitrogen). The H6f represents the N-terminal His6-FLAG tag.

pcDNA-H6f-Nanog_W8A. The mCherry (mCh) gene was amplified from the pBRY-nuclear mCherry-IRES-PURO (Addgene #52409) using the primer sets (43-1F/43-1R) and subcloned into XhoI/XbaI-digested pcDNA3-EGFP (Addgene #13031) to generate pcDNA-mCh. The Nanog gene was amplified from the pEP4 E02S EN2L using the primer sets (43-2F/43-2R) and subcloned into KpnI/XhoI-digested pcDNA-mCh to generate pcDNA-Nanog-mCh. The eight tryptophan residues within the WR domain in the pcDNA-Nanog-mCh were mutated to alanine (W8A) by QuikChange Multi Site-Directed Mutagenesis Kit (Agilent) to generate pcDNA-Nanog_W8A-mCh. First, mutation of the four tryptophan residues (W196, W206, W221, and W231) to alanine were performed using the primer sets (45-5F, 45-6F, 45-7F, and 45-14F) according to the manufacturer's protocol and verified by DNA sequencing. The remaining four tryptophan residues (W201, W216, W226, and W236) were further mutated to alanine in the same method described above using the primer sets (45-10F, 45-15F, 45-16F, and 45-13F). The Nanog containing W8A region was amplified from the pcDNA-Nanog_W8A-mCh using the primer sets (48-1F/48-1R). The vector fragment was amplified from the pcDNA-H6f-Nanog using the primer sets (48-2F/48-2R). Two PCR fragments were ligated using Gibson Assembly Master Mix (NEB) to generate pcDNA-H6f-Nanog_W8A.

pcDNA-H6f-Nanog_ΔWR. To remove WR domain (ΔWR) in the Nanog gene, two fragments were amplified from the pcDNA-Nanog-mCh using the primer sets (45-3F/45-3R and 45-4F/45-4R, respectively) and ligated together using Gibson Assembly Master Mix to generate pcDNA-Nanog_ΔWR-mCh. The Nanog lacking the WR domain (Nanog_ΔWR) region was amplified from the pcDNA-Nanog_ΔWR-mCh using the primer sets (48-1F/48-1R). The vector fragment was amplified from the pcDNA-H6f-Nanog using the primer sets (48-2F/48-2R). Two PCR fragments were ligated using Gibson Assembly Master Mix to generate pcDNA-H6f-Nanog_ΔWR.

pcDNA-Nanog-mCh. The mCherry (mCh) gene was amplified from the pBRY-nuclear mCherry-IRES-PURO (Addgene #52409) using the primer sets (43-1F/43-1R) and subcloned into *Xho*I/*Xba*I-digested pcDNA3-EGFP. The NANOG gene was amplified from the pEP4 E02S EN2L using the primer sets (43-2F/43-2R) and subcloned into *Kpn*I/*Xho*I-digested pcDNA-mCh to generate pcDNA-Nanog-mCh.

pcDNA-Nanog_ΔWR-mCh. To remove WR domain (ΔWR) in the pcDNA-Nanog-mCh, two fragments were amplified from the pcDNA-Nanog-mCh using the primer sets (45-3F/45-3R and 45-4F/45-4R, respectively) and ligated together using Gibson Assembly Master Mix.

XLone-H6f-Nanog-GFP. To construct inducible Nanog expression in the PiggyBac system, the H6f-Nanog region was amplified from the pcDNA-H6f-Nanog using the primer sets (52-1F/52-1R). The vector fragment was amplified from the XLone-GFP (Addgene #96930) using the primer sets (52-2F/52-2R). Two PCR fragments were ligated using Gibson Assembly Master Mix.

XLone-H6f-Nanog_W8A-GFP. The Nanog_W8A region was amplified from the pcDNA-Nanog_W8A-mCh using the primer sets (69-3F/69-3R). The vector fragment was amplified from

the XLone-H6f-Nanog-GFP using the primer sets (69-4F/69-4R). Two PCR fragments were ligated using Gibson Assembly Master Mix.

XLone-Nanog-mCh. The Nanog-mCherry region was amplified from the pcDNA-Nanog-mCh using the primer sets (52-3F/52-3R). The vector fragment was amplified from the XLone-GFP using the primer sets (52-4F/52-2R). Two PCR fragments were ligated using Gibson Assembly Master Mix.

XLone-Nanog_ΔWR-mCh. The Nanog_ΔWR-mCherry region was amplified from the pcDNA-Nanog_ΔWR-mCh using the primer sets (52-3F/52-3R). The vector fragment was amplified from the XLone-GFP using the primer sets (52-4F/52-2R). Two PCR fragments were ligated using Gibson Assembly Master Mix.

pLVX-H6f-Nanog-GFP. To construct inducible Nanog expression in the lentivirus expression system, the H6f-Nanog-GFP region was amplified from the XLone-H6f-Nanog-GFP using the primer sets (110-1F/110-1R). The vector fragment was amplified from the pLVX-Ubc-rtTA-Ngn2:2A:EGFP (Addgene #127288) using the primer sets (110-2F/110-2R). Two PCR fragments were ligated using Gibson Assembly Master Mix. The resulting plasmid was used as a template to amplify two fragments using the primer sets (115-1F/115-1R and 115-2F/115-2R, respectively). Two PCR fragments were ligated using Gibson Assembly Master Mix to generate the pLVX-H6f-Nanog-GFP.

pLVX-H6f-Nanog_W8A-GFP. The H6f-Nanog_W8A-GFP region was amplified from the XLone-H6f-Nanog_W8A-GFP using the primer sets (110-1F/110-1R). The vector fragment was amplified from the pLVX-H6f-Nanog-GFP using the primer sets (110-2F/110-2R). Two PCR fragments were ligated using Gibson Assembly Master Mix.

Construction of Bacterial Expression Plasmids. All generated constructs and mutations were confirmed by DNA sequencing (Eurofins Genomics).

H6Gt-Nanog, H6Gt-Nanog(2-95), H6Gt-Nanog(195-305), H6Gt-Nanog(161-305)_C227S_C251S, and H6Gt-Sox2. To express the N-terminal His6-GB1-TEV (H6Gt) fused proteins in the bacterial system, these five plasmids were custom synthesized by GeneArt Gene Synthesis (ThermoFisher Scientific). The plasmid contains 6XHis tag, solubility tag GB1 (B1 domain of streptococcal protein G) and TEV (Tobacco Etch Virus) protease cleavage site with the pET302/NT-His vector backbone.

pET302-H6Gt-Nanog_WR. To express the N-terminal His6-GB1-TEV (H6Gt) fused Nanog WR domain (residues 195-244), the stop codon (TGA) was introduced to the 245 residue in the H6Gt-Nanog(195-305) by QuikChange Site-Directed Mutagenesis Kit (Agilent) using the primer sets (12-1F/12-1R) according to the manufacturer's protocol.

pET303-Nanog_CTD_2C. The Nanog C-terminal domain (residues 161-305) containing two cysteines (2C) region was amplified from the H6Gt-Nanog(161-305)_C227S_C251S using the primer sets (12-3F/12-3R) and subcloned into XbaI/XhoI-digested pET303/CT-His (Invitrogen).

pET15-NH6t-Nanog_DBD. To express the N-terminal streptavidin-binding Nano tag-His6-TEV (NH6t) fused Nanog DBD (residues 94-162), the Nanog DBD region was amplified from the H6Gt-Nanog using the primer sets (79-4F/79-4R). The vector fragment was amplified from the pET15Nano6HT-SMAD1 (DNASU) using the primer sets (79-3F/79-3R). Two PCR fragments were ligated using Gibson Assembly Master Mix.

pET302-H6Gt-Nanog_CTD_2C_W1357A. The four tryptophan residues (W196, W206, W221, and W231) within the WR domain in the pET303-Nanog_CTD_2C were mutated to alanine using the primer sets (17-15F,17-16F,17-17F, and 17-18F) by QuikChange Multi Site-Directed Mutagenesis Kit (Agilent) according to the manufacturer's protocol and verified by DNA

sequencing. The H6Gt-Nanog_CTD region was amplified from the constructed plasmid as mentioned above using the primer sets (19-F/19-R) and subcloned into SacI/BamHI-digested H6Gt-Nanog(161-305)_C227S_C251S to generate pET302-H6Gt-Nanog_CTD_2C_W1357A.

pET302-H6Gt-Nanog_CTD_2C_W468A. The three tryptophan residues (W216, W226, and W236) within the WR domain in the pET303-Nanog_CTD_2C were also prepared by the same method described in the pET302-H6Gt-Nanog_CTD_2C_W1357A using the primer sets (17-20F,17-21F, and 17-22F).

pET302-H6Gt-Nanog_W8A. The Nanog_W8A region was amplified from the XLone-H6f-Nanog_W8A-GFP using the primer sets (108-4F/108-4R). The vector fragment was amplified from the H6Gt-Nanog using the primer sets (108-5F/108-5R). Two PCR fragments were ligated using Gibson Assembly Master Mix.

pMal-Nanog-Avi-His/BirA. To generate the MBP-NANOG WT with a C-terminal Avitag and histag, the Nanog region was amplified from the H6Gt-Nanog using the primer sets (108-1F/108-1R). The vector fragment was amplified from the pMal-T-Avi-His/BirA (Addgene #102962) using the primer sets (108-2F/108-2R). Two PCR fragments were ligated using Gibson Assembly Master Mix.

pMal-Nanog_W8A-Aiv-His/BirA. For MBP-NANOG W8A construct, the Nanog_W8A region was amplified from the XLone-H6f-Nanog_W8A-GFP using the primer sets (108-3F/108-3R). The vector fragment was amplified from the pMal-T-Avi-His/BirA using the primer sets (108-2F/108-2R). Two PCR fragments were ligated using Gibson Assembly Master Mix.

pET302-H6Gt-GFP. The eGFP gene was amplified from the OCT4-GFP-2A-PURO (Addgene #52379) using the primer sets (9-1F/9-2R), followed by restriction digestion of PCR product with SacI/BamHI, and subcloned into SacI/BamHI-digested H6Gt-Nanog(161-305)_C227S_C251S plasmid vector to generate the h6g-eGFP construct.

pACYC-Skp. To remove trigger factor (TF) region in the pACYC-Skp-TF (Addgene #83921), two fragments were amplified from the pACYC-Skp-TF using the primer sets (109-1F/109-1R and 109-2F/109-2R, respectively) and ligated together using Gibson Assembly Master Mix.

pACYC-H6Gt-Sox2. The H6Gt-Sox2 region was amplified from the H6Gt-Sox2 (GeneArt Gene Synthesis) using the primer sets (109-6F/109-6R). The vector fragment was amplified from the pACYC-Skp-TF using the primer sets (109-4F/109-4R). Two PCR fragments were ligated using Gibson Assembly Master Mix.

Generation of h6f-NANOG WT/W8A-eGFP and NANOG WT/ΔWR-mCherry HEK 293T stable cell lines. To integrate the transposon of XLone-based NANOG constructs (*XLone-H6f-Nanog-GFP*, *XLone-H6f-Nanog_W8A-GFP, XLone-Nanog-mCh*, *XLone-Nanog_ΔWR-mCh*) into the genome of host cells, HEK 293T cells (1x10^6 cells) were nucleofected with XLone constructs (4 μg) and pGLAST-PBase (4 μg) in 100 μL nucleofection solution using a 4D-Nucleofector X Unit (Lonza) according to the manufacturer's protocol. Cells were grown in the DMEM 10% FBS medium and further selected by blasticidin to kill non-nucleofected cells. To induce protein expression, doxycycline (DOX; 2 μg/mL) was added in the cell medium two days after cell passaging.

Generation of h6f-NANOG WT/W8A-eGFP H9 ES stable cell lines. Recombinant lentiviruses were produced by co-transfection of Lenti-X 293T cells (1x10^6 cells in gelatin-coated 10 cm cell culture dish) with lentiviral transfer construct (7 μg), psPAX2 packaging plasmid (7 μg, Addgene #12260), and pMD2.G envelope plasmid (7 μg, Addgene #12259) using polyethylenimine (PEI, 53 μg). Briefly, three plasmids and PEI were diluted with DMEM/F12 medium, left at room

temperature for 20 min, and then added to Lenti-X 293T cells with 10 mL expression medium (DMEM/F12 including 2% FBS). Lentiviruses were harvested 3 days post-transfection, filtered through 0.45 µm-pore size PES filter, and concentrated 100 times using Lenti Concentrator (OriGene).

H9 (WA09) ESCs were cultured in StemFlex medium (Thermo Fisher, A3349401). Before lentiviral infection, colonies were passaged as small clusters using 0.5 mM EDTA in DPBS and plated onto Cultrex coated dishes (Stem Cell Qualified Reduced Growth Factor Basement Membrane Extract, R&D Systems, 3434-010-02) according to manufacturer's recommendation. On the following day, ESCs were infected with a low titer all-in-one lentivirus coding for inducible NANOG-GFP WT or W8A mutant, constitutive reverse-tetracycline trans-activator and puromycin selection. Two days post-infection, cells were selected with 2 µg/ml puromycin (Sigma-Aldrich, P7255-25MG) for 5 days. Expression of NANOG was checked with fluorescence imaging upon addition of 2 µg/ml of doxycycline (DOX; Sigma-Aldrich, D9891-5G). Microscopy imaging and Alkaline Phosphatase (AP) staining were used to monitor mutant ESCs differentiation.

Protein expression from *E. coli.* All protein expression (unless explicitly stated otherwise were performed using the following protocol. Corresponding plasmids were transformed into E. coli BL21 star competent cells (Novagen, Merck KGaA, Darmstadt, Germany). Transformed cells were grown at 37°C in Terrific Broth media containing 100 µg/mL carbenicillin antibiotic until optical density at 600 nm (OD_{600}) reached 0.6. The culture was then transferred to an 18°C incubator shaking at 250 rpm until OD_{600} reached 0.8 to 1.0. Protein expression was induced with 1 mM IPTG, followed by overnight growth at 18°C with shaking at 250 rpm. Cells from the overnight culture were harvested by centrifugation at 8,000 x g.

Protein Purification

HistagGB1(h6g)-fused NANOG constructs. All h6g-NANOG constructs (h6g-NANOG WT/W8A, h6g-NANOG NTD, h6g-NANOG WR, h6g- NANOG CTD and mutants (W1357A,W468A)) were purified with similar protocols. H6g-NANOG WR containing constructs (h6g-NANOG, h6g-WR, h6g-NANOG CTD) were all insoluble (expressed in inclusion bodies). The cell culture pellets were resuspended in denaturing AF lysis buffer (8 M urea, 50 mM Tris, pH 8, 150 mM NaCl). The cells were lysed using a cell homogenizer (Avestin, Ottawa, Canada), with the soluble fraction separated from the cell debris by centrifugation at 18,500 x g for 1 hr. Lysate containing the soluble fraction was filtered using a 0.2 µm filter (Corning). The His-tag fusion protein was purified from the crude protein mixture by immobilized metal-affinity chromatography (IMAC) using batch/gravity method. The lysate was applied to a pre-equilibrated 5 mL HisPur cobalt resin (Thermo Fisher Scientific) followed by extensive washing (20-50 column volumes). The protein was eluted using denaturing lysis buffer supplemented with 200 mM imidazole. The eluted protein was combined with 3 column volumes of 0.1% TFA and acidified to ~ pH 3. The soluble fraction was further purified by reverse-phase HPLC using Zorbax 300SB C3 column (Agilent) and lyophilized. Afterward, the lyophilized His-tag proteins were dissolved in 6 M Gdn HCl and diluted ten times with 20 mM Tris pH 8 before the addition of TEV protease (1 mg : 25 mg TEV:protein concentration ratio). The samples were incubated overnight at room temperature (RT) with rotation. Urea was added up to 6 M to dissolve any precipitates before passing through the cobalt resin to separate the uncleaved proteins. The cleaved, untagged proteins were purified again by reverse-phase HPLC using a similar method described above.

NANOG CTD (untagged). Cell pellets were initially lysed in AF buffer (50 mM Tris, pH 8, 150 mM NaCl without urea). After centrifugation, the cell pellet was resuspended in denaturing AF lysis buffer and centrifuged at 18,500 xg for 1 hr to separate cell debris. The supernatant was mixed with 3 volumes of 0.1% TFA and purified by reverse-phase HPLC using Zorbax 300SB C3 column (Agilent). Fractions with impurities were purified again with 2-3 rounds of reverse-phase HPLC. Purified fractions were lyophilized and stored at -80°C.

NANOG DBD (Nh6t-Nanog DBD). Cell culture pellets were resuspended in denaturing lysis buffer (6 M urea, 1 mM 2-mercaptoethanol, 0.5 M NaCl, 109 mM NaPhos, pH 8). The resuspended pellets were lysed using a cell homogenizer (Avestin, Ottawa, Canada), with the soluble fraction separated from the cell debris by centrifugation at 18,500 x g. The lysate containing the soluble fraction was filtered using a 0.2 µm filter (Corning). The His-tagged fusion protein was purified from the crude protein mixture by IMAC batch/gravity method. The lysate was applied to a pre-equilibrated 5 mL HisPur cobalt resin (Thermo Fisher Scientific) followed by extensive washing (20-50 column volumes). The protein was eluted using denaturing lysis buffer supplemented with 200 mM imidazole. The eluted protein was combined with 3 column volumes of 0.1% TFA, and acidified to ~ pH 3. The soluble fraction was further purified by reverse-phase HPLC using Zorbax 300SB C3 column (Agilent). Pure fractions were then combined and dialyzed against deionized distilled water (dH_2O). Afterwards, the protein solution adjusted to ~ pH 6-7 with 1 M Tris, pH 8 (final concentration ~10-20 mM). TEV protease was then added (1 mg : 25 mg TEV:protein concentration ratio), and the sample incubated overnight with rotation. The cleaved, untagged proteins were subsequently purified by HPLC. After dialysis against dH_2O, the samples were flash frozen and stored at -80°C.

Full-length (FL) MBP-NANOG WT and W8A (with C-terminal Histag). Cell pellets (from 10 mL *E. coli* culture) were resuspended in 700 µl CEB buffer (10 mM Tris, pH 7.4, 10 mM NaCl, 1%(v/v) Triton X-100, 10%(v/v) glycerol, 0.5% sodium deoxycholate, 0.1% SDS) and lysed using a handheld sonicator (Microson; 3 x 60 short pulses of 30% power) and incubated on ice for 1 hr. The cell lysates were centrifuged for 30 min at 17,000 x g. The supernatant was added with 10% SDS (final concentration of 1%), mixed with the cobalt beads (Thermo Scientific), and rotated for 2 hr at RT. The 1% SDS condition was necessary for MBP-NANOG WT to bind to the resin). The beads were extensively washed with 10-fold column volumes of CEB buffer, then with 40-fold column volumes of W2 buffer (40 mM Tris, 120 mM NaCl, 1%(v/v) Triton x-100) and eluted with W2 buffer with 200 mM Imidazole, pH 8. The eluate was also concentrated in 100 kD small spin concentrators (Corning).

H6g-NANOG WT/W8A with Skp molecular chaperone. The pair of plasmids, h6g-NANOG and pACYC-Skp or pET302-h6g-Nanog_W8A and pACYC-Skp, were co-transformed into competent *E.coli* BL21 star (DE3) and plated onto LB agar plates containing carbenicillin (100 µg/mL) and chloramphenicol (36 µg/mL). Transformants were grown in Terrific Broth in the presence of carbenicillin and chloramphenicol to an OD_{600} of 1.0 at 37°C. Protein expression was induced by the addition of 1 mM IPTG and 0.1 mM $ZnSO_4$ and growth was continued overnight at 18°C. Cell pellets from 10 mL *E. coli* culture were resuspended in 1 mL High salt buffer (25 mM Hepes, 1 M NaCl, 5 mM Imidazole, pH 7.6), lysed using a handheld sonicator (60 short pulses of 20% power) and incubated on ice for 30 min. The cell lysates were centrifuged for 30 min at 17,000 x g. The supernatant was passed through the Talon resin (Thermo Fisher). The beads were extensively washed with 50-fold column volumes of High salt buffer and eluted with the same buffer with 200 mM Imidazole.

FL SOX2. Cell culture pellet was resuspended in denaturing lysis buffer (8 M urea, 850 mM NaCl, 50 mM Tris, pH 8), lysed, and centrifuged. The supernatant was subsequently passed

through an IMAC column with cobalt resin. After 20 column volumes of washing with the lysis buffer, the protein was eluted using the same buffer supplemented with 200 mM imidazole. The eluted protein was concentrated and then diluted six-fold with refolding buffer (PBS supplemented with 500 mM NaCl, 5%(v/v) glycerol, and 0.1%(v/v) Tween-20). The h6g fusion tags from h6g-SOX2 proteins were cleaved (1:20 TEV:protein w/w ratio) overnight at 4°C. Cobalt resin was used to remove h6GB1 and uncleaved proteins; Q Sepharose beads (GE Healthcare) were subsequently used to remove excess DNA. The flow-through was mixed with TFA to a final concentration of 0.2% TFA and finally purified by C3 reverse phase HPLC. Purified fractions were lyophilized and stored at -80°C.

h6g-eGFP and h6g-mCherry. Cell pellets were resuspended and lysed in AF buffer (50 mM Tris, 150 mM NaCl, pH 8). The fluorescent proteins were subsequently purified using IMAC batch/gravity method. The beads were extensively washed with 50-fold column volumes and the protein was eluted with 200 mM Imidazole. The eluate was dialyzed in 20 mM Tris, pH 7.5 and snap frozen at -80°C until further use.

Purification of h6f-NANOG WT/W8A-eGFP from HEK 293T cells. HEK 293T cell pellets (~5 x 10^7 cells) were resuspended in RIPA buffer (50 mM Tris, pH 7.5, 150 mM NaCl, 0.5% sodium deoxycholate, 1% Triton X-100) supplemented with 150U benzonase (EMD Millipore), 1x protease inhibitor cocktail (GenDepot), 0.5 mM $CaCl_2$, and 2.5 mM $MgCl_2$. The cells were lysed with 20 pulses of 10% power by handheld sonicator (Microson) and incubated on ice overnight. The cell lysates were centrifuged for 30 min at 17,000 x g. Additional NaCl and Imidazole (up to final 1 M and 10 mM concentration, respectively) for efficient binding to the cobalt resin (Talon; Thermo Fisher). The beads were rotated for 2 hr at 4°C. The beads were extensively washed with 50-fold column volumes of RIPA buffer and eluted with RIPA-IMI buffer (40 mM Tris, pH 7.5, 120 mM NaCl, 0.4% sodium deoxycholate, 0.8% Triton X-100, 200 mM Imidazole). The samples were dialyzed against RIPA-IMI buffer and quantified by h6g-eGFP fluorescence calibration (both in gel and using Tecan microplate reader). The samples are stored on ice and used immediately (within 2-3 days) for EMSA and FCS experiments.

Preparation of unlabeled and fluorescently labeled DNA

GATA6 DNA. To prepare differentially fluorescently labeled DNA (AF488 dsDNA, AF647 dsDNA, AF488/AF647 dsDNA), fluorescent labeling was performed on single strand DNA (ssDNA) prior to annealing. Briefly, *GATA6* single strand DNA oligos with 5' amino modified C6 (IDT) were purified by ethanol precipitation and labeled with a 10-fold molar excess of dye (Alexa Fluor 488 or 647 NHS ester; Invitrogen) to DNA. The labeling reactions were performed at 30°C with 30 to 60 min incubation. The corresponding fluorescent or unlabeled ssDNA pairs were annealed using Proflex PCR machine (Life Technologies) with slow temperature decrement from 95°C to RT in ~4 hours. The fluorescent conjugated *GATA6* dsDNA samples were then ethanol precipitated. DNA pellets were dissolved in 0.1 M TEAA (triethylammonium acetate) at pH 7, and reverse-phase HPLC (with 10-60% gradient of 0.1 M TEAA pH 7/50% ACN with Zorbax SB C8 column) was performed to separate excess unconjugated dyes, ssDNA and dsDNA.

pγSat satellite DNA (257 bp). The mouse γ-satellite repeats (257 bp) were amplified by PCR from the *pγSat* (Addgene #39238) using the primer sets (56-5F/58-1R-AF647). Prior to the PCR reaction, the primer 58-1R was labeled with AF647 at the 5'-end of the primer. Briefly, the 58-1R primer (214 µg, lyophilized) was added to 40 µL of 0.2 M sodium phosphate, pH 8.0 and 20 µL of 2 mg/mL-Alexa Fluor 647 NHS Ester (ThermoFisher Scientific), followed by incubation at 30°C

for 1 hr. The labeled primer was purified by ethanol precipitation. The fluorescently-labeled *pγSat*-AF647 was purified QIAquick PCR Purification Kit (Qiagen).

NANOG promoter (404 bp). The human *Nanog* promoter (404 bp) was amplified by PCR from the pNanog-Luc (Addgene #25900) using the primer sets (hNan-F2/hNan-R2). Purified PCR products (lyophilized, 7-8 μg) were added to 13 μL nuclease-free water and incubated at 42°C for 5 min to dissolve DNA completely. After adding 2 μL of 7.5% $NaHCO_3$ and 5 μL of 2 mg/mL-Alexa Fluor 647 NHS Ester (ThermoFisher Scientific), the reaction mixtures were further incubated at room temperature for 2 hr in the dark. The labeled DNA was added to 160 µL of nuclease-free water and 20 µL of 3 M NaOAc (pH 5.2), mixed well, and then purified with QIAquick PCR Purification Kit (Qiagen).

Preparation of fluorescently-labeled NANOG constructs and SOX2

NANOG and SOX2. For FCS experiments, untagged and purified NANOG and SOX2 were labeled with Alexa Fluor 488 maleimide (Thermo Fisher Scientific) using standard methods described previously[25]. Briefly, the lyophilized proteins were resuspended in 7.2 M Gdn HCl, $\alpha\beta\gamma$ buffer (10 mM OAc,10 mM glycine, 10 mM NaPhos, 200 mM NaCl, pH 7.2) and the appropriate concentrations were mixed with Alexa Fluor 488 maleimide at a 1:1 protein to dye molar ratio. The reaction was incubated for 1 hr at RT. The fluorescently labeled samples were then diluted 4-fold with 0.2% TFA/20% ACN centrifuged to remove precipitates and purified by reverse-phase HPLC (with 10-60% gradient of 0.1% TFA/100% ACN) using a Zorbax SB C3 column (Agilent).

NANOG CTD (C221S, C251S, 185C and 243C). For smFRET experiments, the lyophilized protein was resuspended in 7.2 M Gdn HCl, $\alpha\beta\gamma$ buffer, and the appropriate concentration was mixed with Alexa Fluor 488 and Alexa Fluor 594 maleimide (1:1:3 molar ratio, respectively). The reaction was allowed to proceed for 1 hr at RT and subsequently purified by reverse phase HPLC as described above. Purified fluorescently labeled samples were lyophilized and stored at -80°C.

Protein and DNA Concentration Determination. Protein concentrations of recombinant NANOG constructs (except for MBP-NANOG constructs) were calculated based on their UV absorbance extinction coefficients at 280 nm (based on Tyr and Trp absorbance[54]). H6g-eGFP and h6g-mCherry concentrations were determined based on UV absorbance extinction coefficients at 488 nm of 56,000 $M^{-1}cm^{-1}$ and 587 nm of 72,000 $M^{-1}cm^{-1}$, respectively. Fluorescent NANOG concentrations were determined using calibrations of known GFP/mCherry concentrations using ImageJ quantitation of SDS-PAGE gels (*e.g.* Extended Data Fig. 5; where the proteins were not heated or denatured to retain native fluorescence), or using a fluorescence-based microplate reader (Tecan). MBP-NANOG protein concentrations were based on BSA calibration using stain-free detection (tryptophan detection only in stain-free SDS-PAGE gels; Bio-rad). The number of tryptophans present in BSA, NANOG WT, W8A constructs is 3, 20, and 12, respectively. Band intensities were quantified by ImageJ.

Fluorescent DNA concentrations were measured using the extinction coefficients of the Alexa Fluor 488 and 647 dyes. Fluorescent labeling efficiencies were calculated using the corrected extinction coefficients based on the manufacturer's protocol (Invitrogen).

Additional supplementary figure:

Supplementary Figure 1. (Related to Extended Data Figure 7) | Auto and Cross-Correlation FCCS measurements of *Gata6-DNA* (AF488/AF647) with 0-875 nM NANOG WT and W8A. Individual normalized auto and cross-correlation functions (measurements, symbols; FCCS fits and residuals, lines).

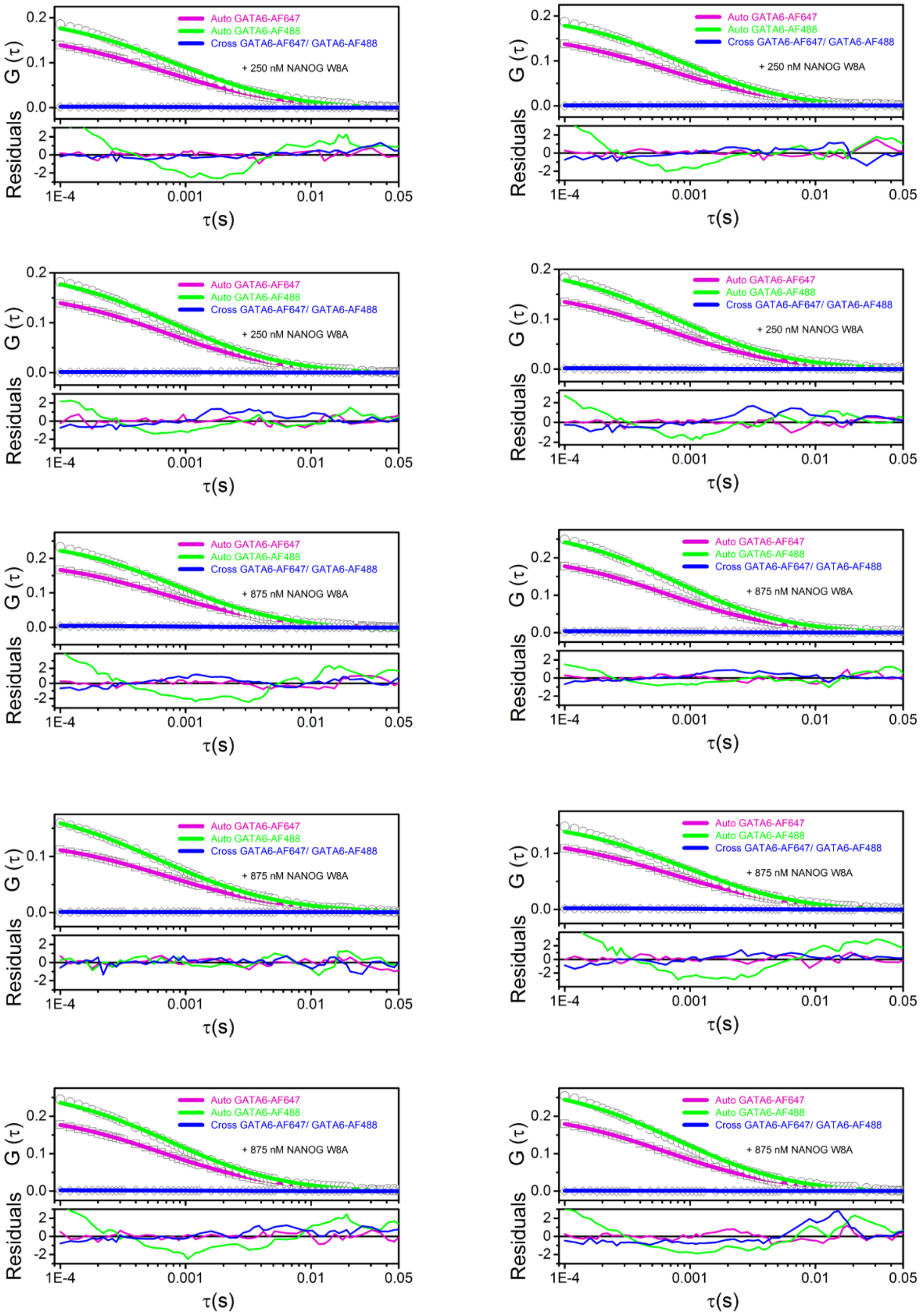

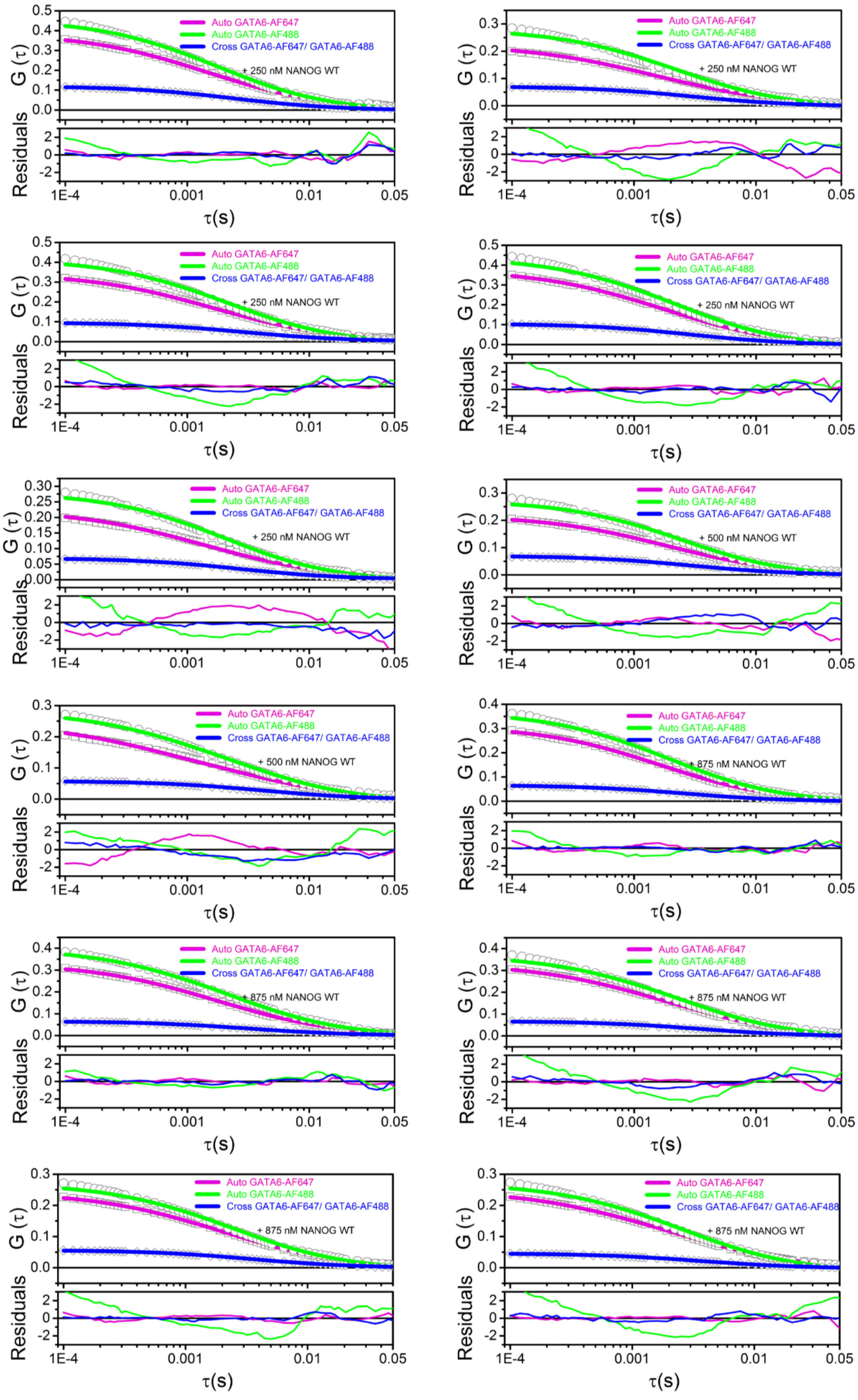
Auto GATA6-AF647
Auto GATA6-AF488
Cross GATA6-AF647/ GATA6-AF488
+ 250 nM NANOG WT
+ 500 nM NANOG WT
+ 875 nM NANOG WT
G (τ)
Residuals
τ(s)
1E-4
0.001
0.01
0.05

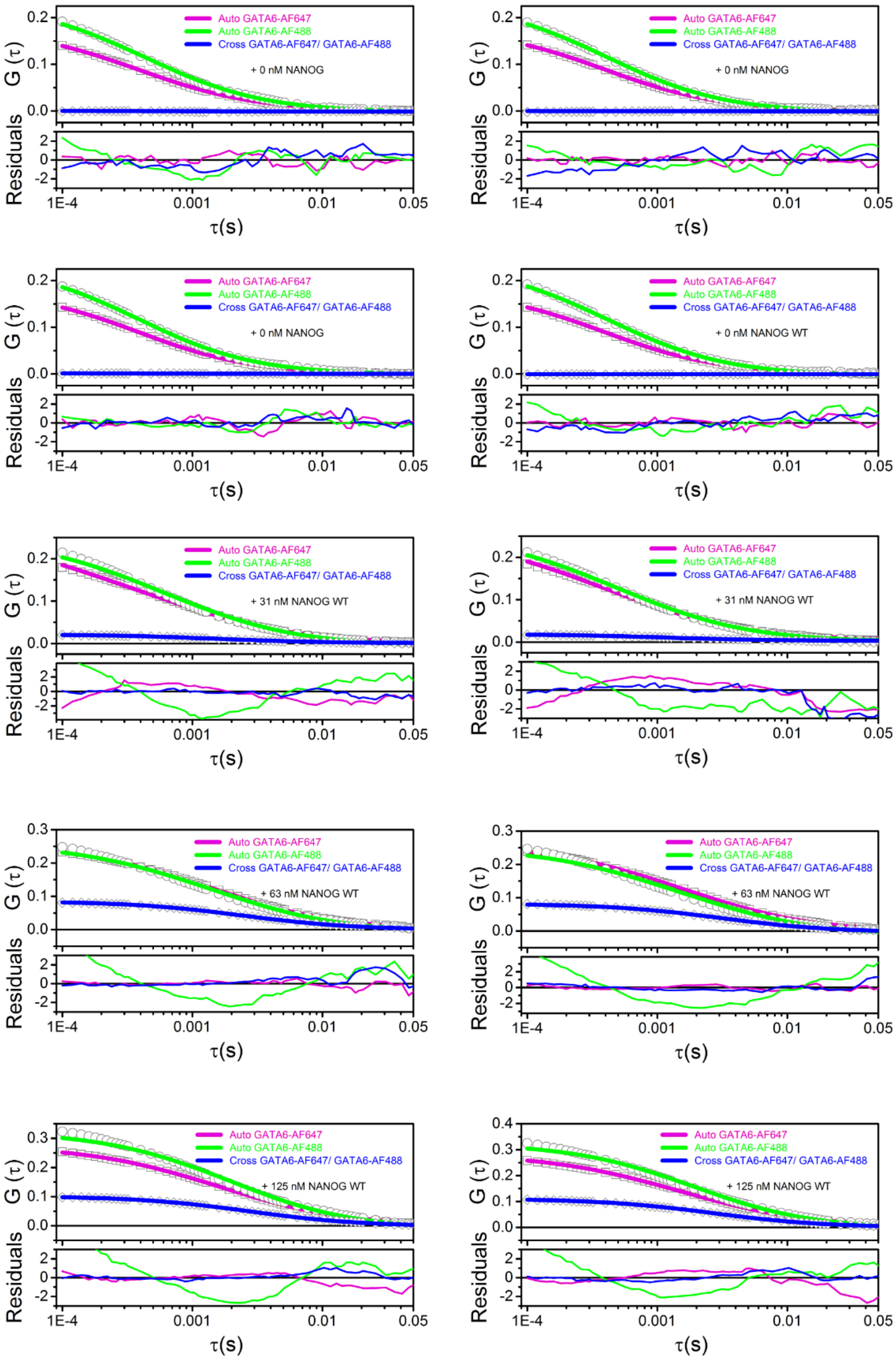

Auto GATA6-AF647
Auto GATA6-AF488
Cross GATA6-AF647/ GATA6-AF488
+ 0 nM NANOG
+ 0 nM NANOG WT
+ 31 nM NANOG WT
+ 63 nM NANOG WT
+ 125 nM NANOG WT
G (τ)
Residuals
τ(s)
1E-4
0.001
0.01
0.05
