# Supplementary material for: NANOG prion-like assembly mediates DNA bridging to facilitate chromatin reorganization and activation of pluripotency
Source: Nat Cell Biol. 2022 Apr 28;24(5):737–47. doi: 10.1038/s41556-022-00896-x (PMC9106587; doi:10.1038/s41556-022-00896-x)
Supplement: Supplementary file 2 — Reporting Summary [file 41556_2022_896_MOESM2_ESM.pdf]

nature research

Corresponding author(s): Dr. Josephine Ferreon

Last updated by author(s): Mar 11, 2022

# Reporting Summary

Nature Research wishes to improve the reproducibility of the work that we publish. This form provides structure for consistency and transparency in reporting. For further information on Nature Research policies, see our Editorial Policies and the Editorial Policy Checklist.

## Statistics

For all statistical analyses, confirm that the following items are present in the figure legend, table legend, main text, or Methods section.

| n/a | Confirmed | |
|---|---|---|
| ☐ | ☒ | The exact sample size (*n*) for each experimental group/condition, given as a discrete number and unit of measurement |
| ☐ | ☒ | A statement on whether measurements were taken from distinct samples or whether the same sample was measured repeatedly |
| ☐ | ☒ | The statistical test(s) used AND whether they are one- or two-sided<br>*Only common tests should be described solely by name; describe more complex techniques in the Methods section.* |
| ☒ | ☐ | A description of all covariates tested |
| ☒ | ☐ | A description of any assumptions or corrections, such as tests of normality and adjustment for multiple comparisons |
| ☐ | ☒ | A full description of the statistical parameters including central tendency (e.g. means) or other basic estimates (e.g. regression coefficient) AND variation (e.g. standard deviation) or associated estimates of uncertainty (e.g. confidence intervals) |
| ☐ | ☒ | For null hypothesis testing, the test statistic (e.g. *F*, *t*, *r*) with confidence intervals, effect sizes, degrees of freedom and *P* value noted<br>*Give P values as exact values whenever suitable.* |
| ☒ | ☐ | For Bayesian analysis, information on the choice of priors and Markov chain Monte Carlo settings |
| ☒ | ☐ | For hierarchical and complex designs, identification of the appropriate level for tests and full reporting of outcomes |
| ☒ | ☐ | Estimates of effect sizes (e.g. Cohen's *d*, Pearson's *r*), indicating how they were calculated |

*Our web collection on statistics for biologists contains articles on many of the points above.*

## Software and code

Policy information about availability of computer code

| | |
|---|---|
| Data collection | For microscopic imaging, Zen 2.3, Fiji (ImageJ 1.53f51), LSM780/880 laser-scanning confocal microscope software and Nikon NIS software. For smFRET, FCS,FCCS data, we use VistaVision (64) 4.2.220.0. Details of data collection are described in methods.<br><br>We generated ChIP-Seq and Hi-C 3.0 data in HEK293T cell line using Illumina NextSeq 550 platform. The data was demultiplexed using bcl2fastq(v2.2.0). |
| Data analysis | For data analysis, we used OriginPro 2020 and VistaVision (64) 4.2.220.0. For microscopic imaging, Fiji (ImageJ 1.53f51). Details of data analysis were described in the manuscript.<br><br>NGS data software we used: Trimmomatic(v0.36), Bowtie2(v2.2.9), samtools(v1.9), MACS2(v2.2.7), Bedtools(v2.1.0), HiC-Pro(v2.8.10,https://github.com/nservant/HiC-Pro), cooler(https://github.com/open2c/cooler), cooltools (https://github.com/open2c/cooltools), coolpup.py(https://github.com/open2c/coolpuppy), deeptools(v3.5.0), IGV(v2.8.13). |

For manuscripts utilizing custom algorithms or software that are central to the research but not yet described in published literature, software must be made available to editors and reviewers. We strongly encourage code deposition in a community repository (e.g. GitHub). See the Nature Research guidelines for submitting code & software for further information.

## Data

Policy information about availability of data

All manuscripts must include a data availability statement. This statement should provide the following information, where applicable:

- Accession codes, unique identifiers, or web links for publicly available datasets
- A list of figures that have associated raw data
- A description of any restrictions on data availability

Source data for plots, raw data for counts and intensity measurements, and uncropped gel images generated in this study are provided in a Source Data file and Supplementary Information. All raw and processed high-throughput sequencing data generated in this study have been deposited to GEO with accession number: GSE190567. Additional information on sequencing data reported in this paper is available from the corresponding authors upon request.

# Field-specific reporting

Please select the one below that is the best fit for your research. If you are not sure, read the appropriate sections before making your selection.

☒ Life sciences ☐ Behavioural & social sciences ☐ Ecological, evolutionary & environmental sciences

For a reference copy of the document with all sections, see nature.com/documents/nr-reporting-summary-flat.pdf

# Life sciences study design

All studies must disclose on these points even when the disclosure is negative.

| | |
|---|---|
| Sample size | No statistical method was used to predetermine sample size. The sample sizes were ~50-100 cells (each biological replicate) for the cell imaging experiments, consistent with those reported in the literature. |
| Data exclusions | There were no data exclusions. |
| Replication | All attempts at replication were successful. There were 2-3 independent replicate experiments for in vitro and in cell studies. |
| Randomization | No randomization was explicitly performed because analysis is required, consistent with typical practice in biophysical experiments. |
| Blinding | Blinding was not performed for most experiments because analysis is required, consistent with typical practice in biophysical experiments. However, for some FCCS data experiments, blinding was performed because the data collector have no information on the samples. |

# Reporting for specific materials, systems and methods

We require information from authors about some types of materials, experimental systems and methods used in many studies. Here, indicate whether each material, system or method listed is relevant to your study. If you are not sure if a list item applies to your research, read the appropriate section before selecting a response.

### Materials & experimental systems

| n/a | Involved in the study | |
|---|---|---|
| ☐ | ☒ | Antibodies |
| ☐ | ☒ | Eukaryotic cell lines |
| ☒ | ☐ | Palaeontology and archaeology |
| ☒ | ☐ | Animals and other organisms |
| ☒ | ☐ | Human research participants |
| ☒ | ☐ | Clinical data |
| ☒ | ☐ | Dual use research of concern |

### Methods

| n/a | Involved in the study | |
|---|---|---|
| ☐ | ☒ | ChIP-seq |
| ☒ | ☐ | Flow cytometry |
| ☒ | ☐ | MRI-based neuroimaging |

## Antibodies

| | |
|---|---|
| Antibodies used | Anti-Nanog, 1:200 Santa Cruz sc-374103; Anti-GAPDH, 1:6000 Millipore CB1001; Anti-Mouse IgG, 1:1000, Cell Signaling #7076)<br><br>For ChIP-seq experiments, the antibodies (3 ug) we used are described in the Methods section, including commercially available antibodies for NANOG (R&D Systems, AF1997-SP, Polyclonal Goat IgG) and H3K27ac (Abcam, AB4927, Rabbit polyclonal antibody). We use 3ug antibody for each ChIP-seq. |
| Validation | Anti-Nanog was validated by WB with purified Nanog protein, and validated that it does not react with Oct4 and Sox2. |

Validation

All antibodies for ChIP-seq we used here have been validated by manufacturers. Furthermore, these antibodies have been widely used in the human genomic studies; such as PMID:30122536, PMID:33828098 and PMID:33915080.

# Eukaryotic cell lines

Policy information about cell lines

| | |
|---|---|
| Cell line source(s) | The HEK 293T cells were obtained from ATCC; H9 ESC from WiCell and Lenti-X 293T from TaKaRa Bio. |
| Authentication | The cell lines were not authenticated. Authentication came from the source. |
| Mycoplasma contamination | All cells used in this study tested negative for mycoplasm contamination. |
| Commonly misidentified lines (See ICLAC register) | No commonly misidentified lines were used in the study |

# ChIP-seq

## Data deposition

☒ Confirm that both raw and final processed data have been deposited in a public database such as GEO.

☒ Confirm that you have deposited or provided access to graph files (e.g. BED files) for the called peaks.

| | |
|---|---|
| Data access links *May remain private before publication.* | GSE190567 (https://www.ncbi.nlm.nih.gov/geo/query/acc.cgi?acc=GSE190567) |
| Files in database submission | all RAW FASTQ, BIGWIG and mcool were uploaded to NCBI GEO. |
| Genome browser session (e.g. UCSC) | https://genome.ucsc.edu/s/qicy/NANOG |

## Methodology

Replicates

We did two replicates for NANOG ChIP-Seq experiments; two replicates for H3K27ac in 293T with overexpressing NANOG WT or W8A.

Sequencing depth

HEK293T-NANOG_WT_H3K27ac_ChIP-Seq_rep1,ChIP-seq, overexpressing NANOG WT, H3K27ac,biological replicate1, 16286301
HEK293T-NANOG_WT_H3K27ac_ChIP-Seq_rep2,ChIP-seq, overexpressing NANOG WT, H3K27ac,biological replicate2, 10863588
HEK293T-NANOG_W8A_H3K27ac_ChIP-Seq_rep1,ChIP-seq, overexpressing NANOG W8A,H3K27ac,biological replicate1, 18761363
HEK293T-NANOG_W8A_H3K27ac_ChIP-Seq_rep2,ChIP-seq, overexpressing NANOG W8A,H3K27ac,biological replicate2, 9604136
HEK293T-NANOG_WT_NANOG_ChIP-Seq_rep1,ChIP-seq, overexpressing NANOG WT,NANOG,biological replicate1, 18455755
HEK293T-NANOG_WT_NANOG_ChIP-Seq_rep2,ChIP-seq, overexpressing NANOG WT,NANOG,biological replicate2, 24,826,386
HEK293T-NANOG_W8A_NANOG_ChIP-Seq_rep1,ChIP-seq, overexpressing NANOG W8A ,NANOG,biological replicate1, 28767501
HEK293T-NANOG_W8A_NANOG_ChIP-Seq_rep2,ChIP-seq, overexpressing NANOG W8A ,NANOG,biological replicate2, 25916080

Antibodies

ChIP-seq experiments, The antibodies we used are described in this study, including NANOG (R&D Systems AF1997) and H3K27ac (Abcam, AB4927); 3ug antibodies were used for each ChIP-seq.

| | |
|---|---|
| Peak calling parameters | MACS2 (v2.2.7) was used to call ChIP-Seq peaks with --nomodel and --qvalue 0.01 parameter. |
| Data quality | fastqc was used to check raw fastq quality. |
| Software | Trimmomatic(v0.36), Bowtie2(v2.2.9) and MACS2(v2.2.7) |
