# Supplementary figures and images for: NANOG prion-like assembly mediates DNA bridging to facilitate chromatin reorganization and activation of pluripotency
Source: Nat Cell Biol. 2022 Apr 28;24(5):737–47. doi: 10.1038/s41556-022-00896-x (PMC9106587; doi:10.1038/s41556-022-00896-x)

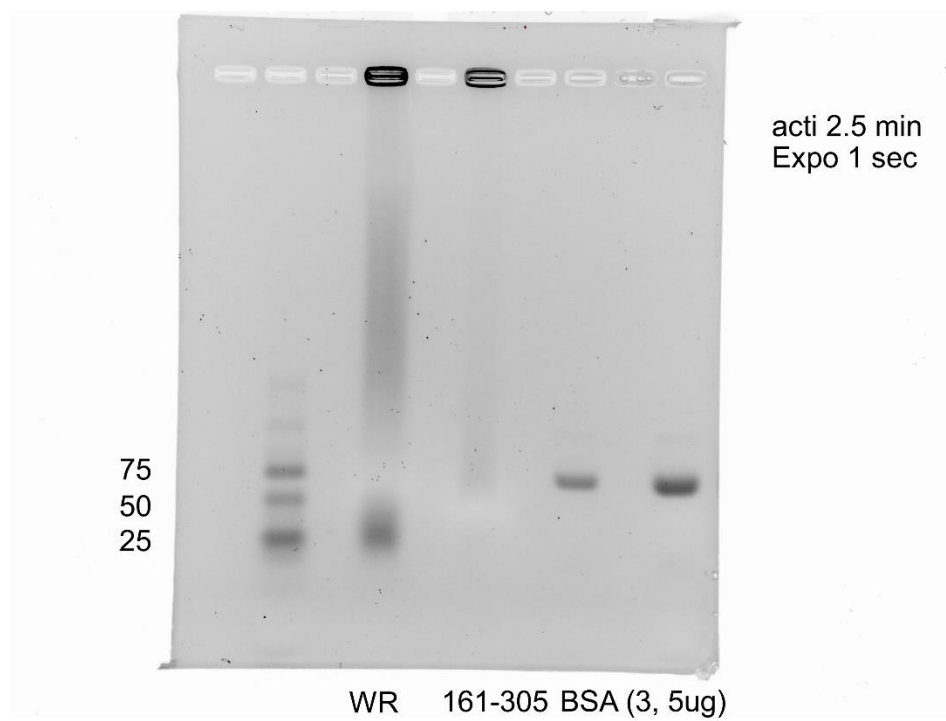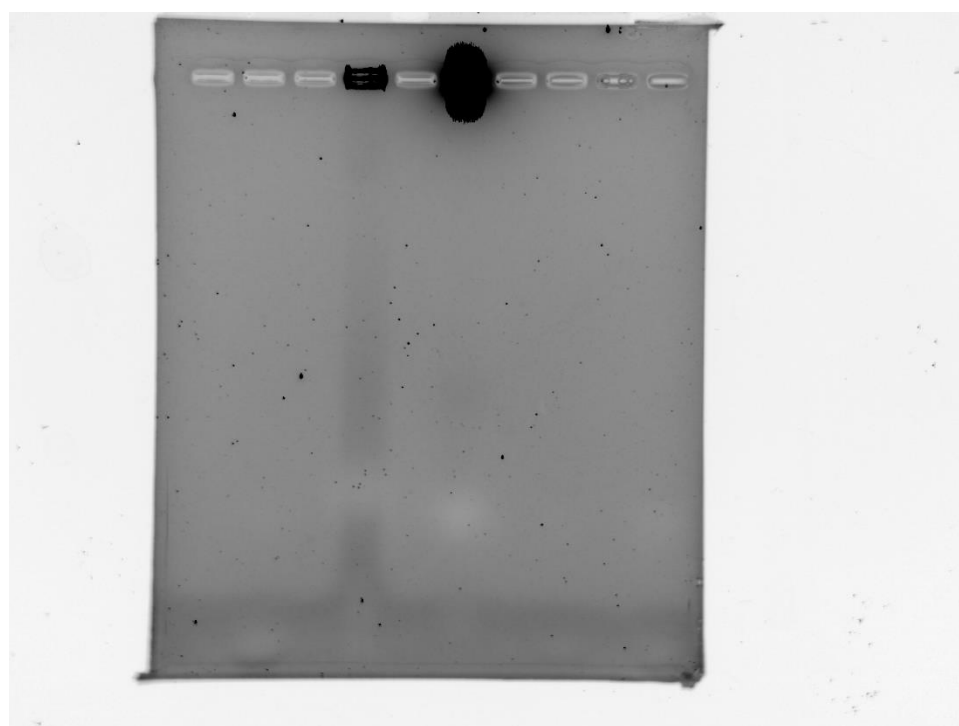

**Figure 2f.** Gels are as in paper.

Supplement: Source Data Fig. 2 — Unprocessed gels. [file 41556_2022_896_MOESM9_ESM.pdf]

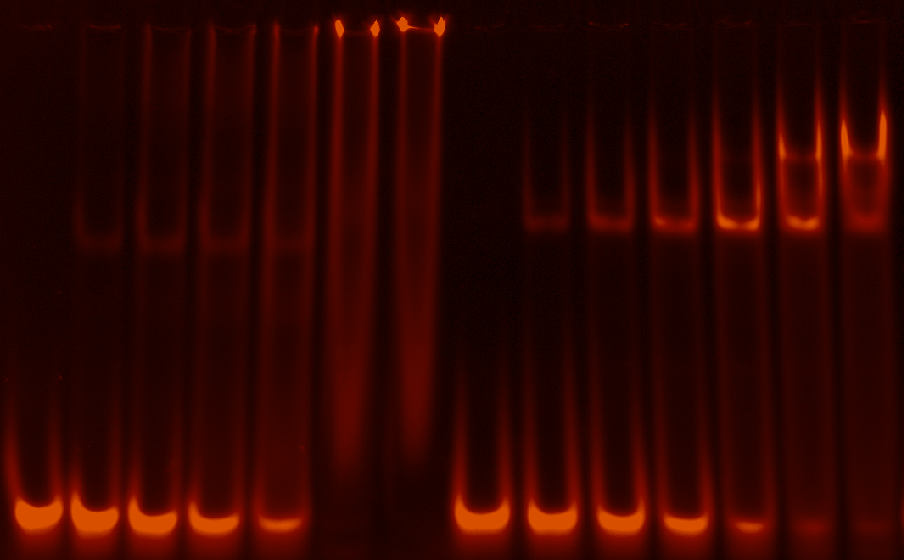

Supplement: Source Data Fig. 4 — Unprocessed gels. [file 41556_2022_896_MOESM11_ESM.tif]

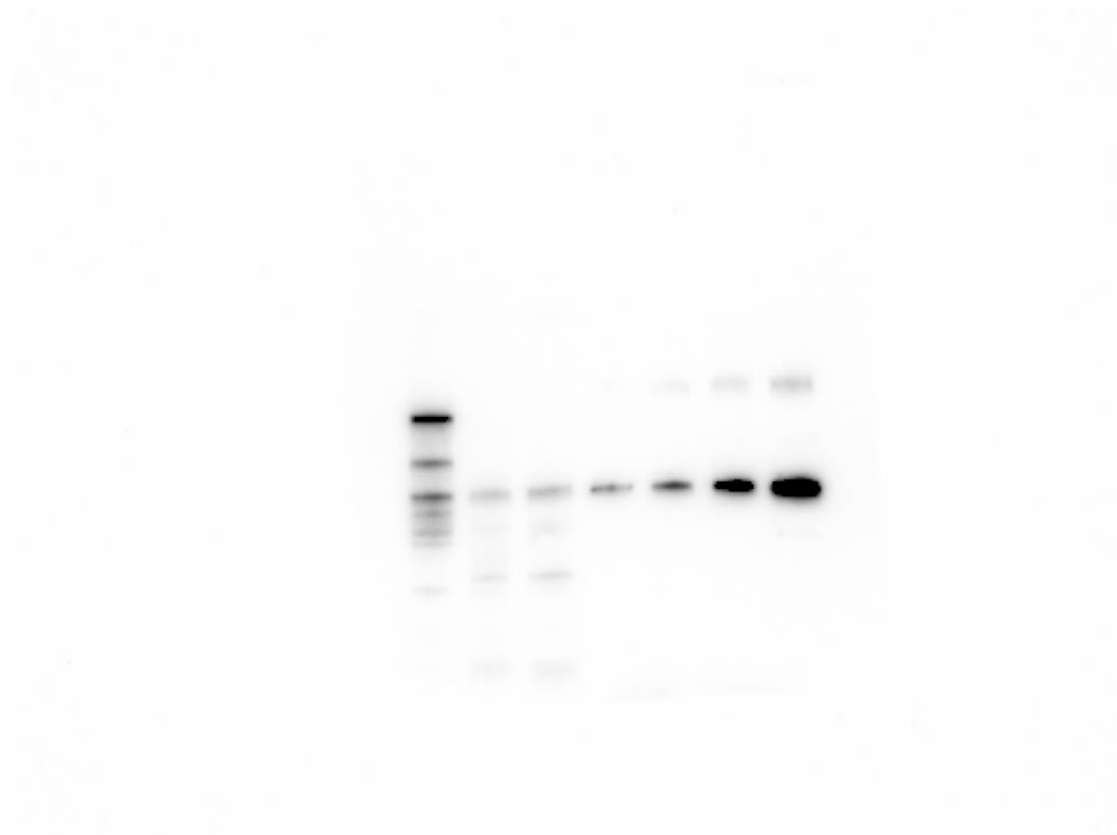

**Extended Data Fig. 4e.** Exact gel as in paper.

Supplement: Source Data Extended Data Fig. 4 — Unprocessed western blot. [file 41556_2022_896_MOESM19_ESM.pdf]
