# Supplementary material for: NANOG prion-like assembly mediates DNA bridging to facilitate chromatin reorganization and activation of pluripotency
Source: Nat Cell Biol. 2022 Apr 28;24(5):737–47. doi: 10.1038/s41556-022-00896-x (PMC9106587; doi:10.1038/s41556-022-00896-x)
Supplement: Source Data Fig. 3 — Unprocessed western blots. [file 41556_2022_896_MOESM10_ESM.pdf]

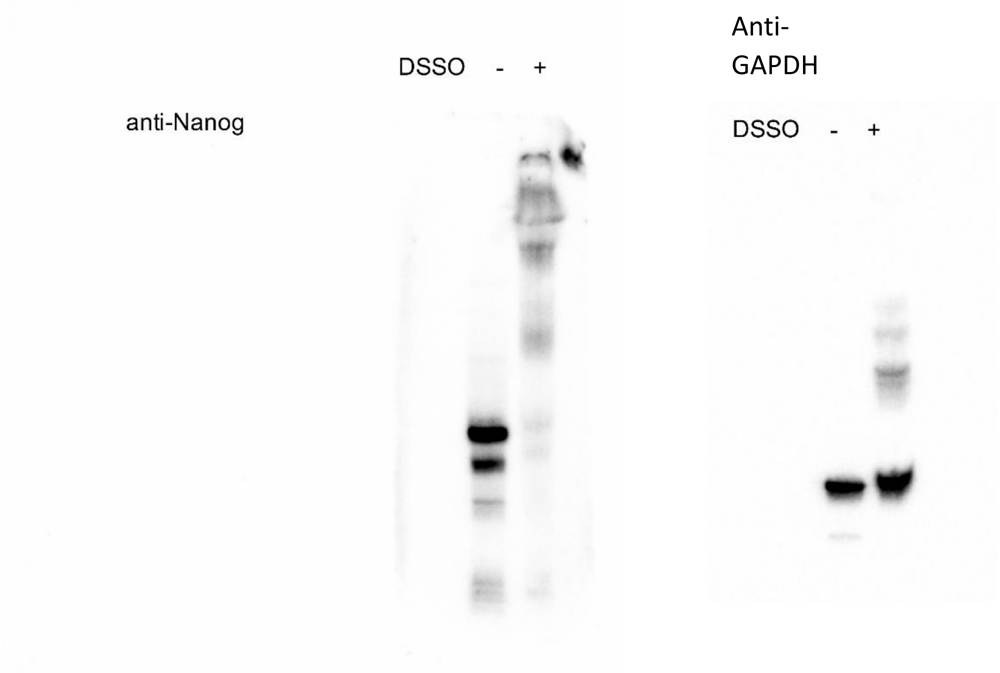

Figure 3a. Left gel corresponds to top figure in paper. Right gel corresponds to bottom figure in paper.

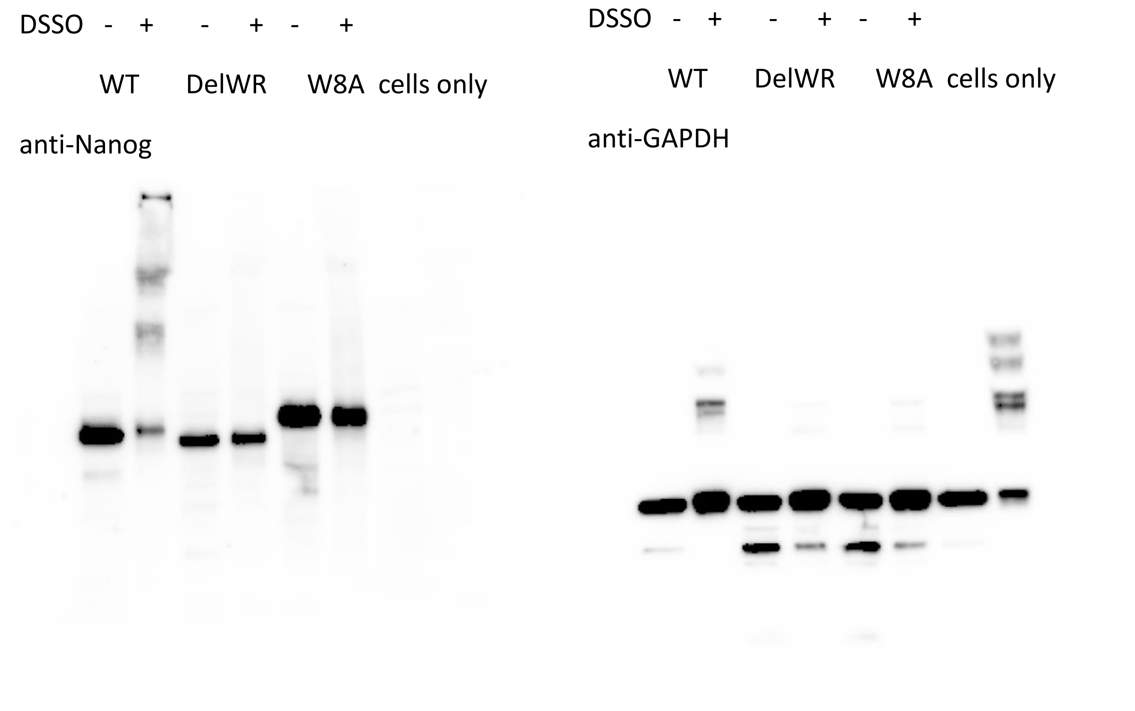

Figure 3b. Left gel corresponds to Lanes 1-7 top figure in paper. Right gel corresponds to Lanes 1-7 bottom figure in paper**.**

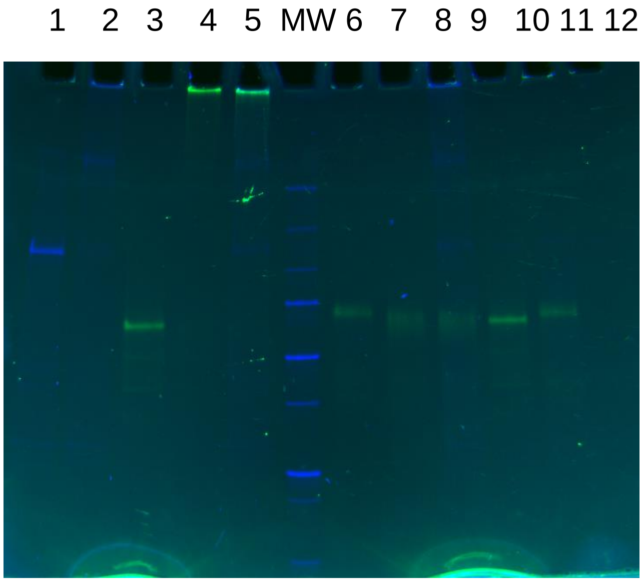

Figure 3e. Lanes 3-4 for WT and lanes -7 for W8A DSSO crosslinking in paper.
