# Supplementary material for: NANOG prion-like assembly mediates DNA bridging to facilitate chromatin reorganization and activation of pluripotency
Source: Nat Cell Biol. 2022 Apr 28;24(5):737–47. doi: 10.1038/s41556-022-00896-x (PMC9106587; doi:10.1038/s41556-022-00896-x)
Supplement: Source Data Extended Data Fig. 2 — Unprocessed gels. [file 41556_2022_896_MOESM18_ESM.pdf]

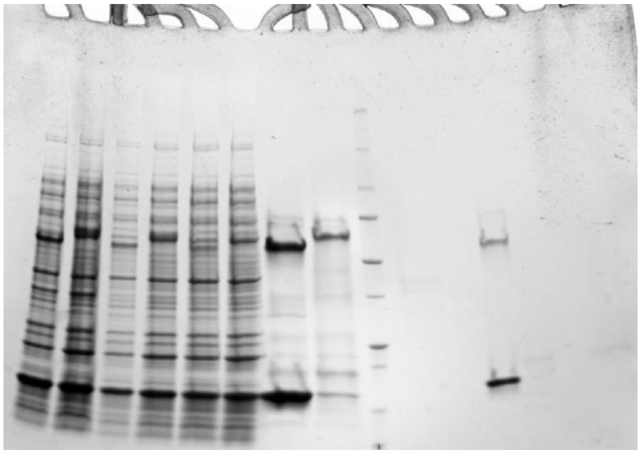

Extended Data Fig. 2a. Lanes 1-10 correspond to same lanes in paper.

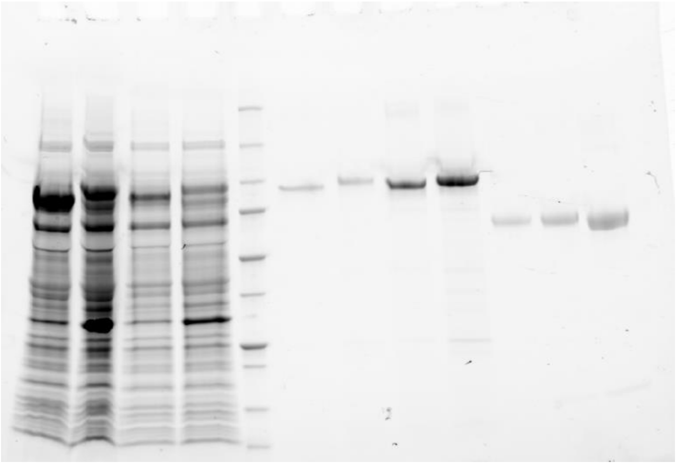

Extended Data Fig. 2b. Exact gel as in paper.

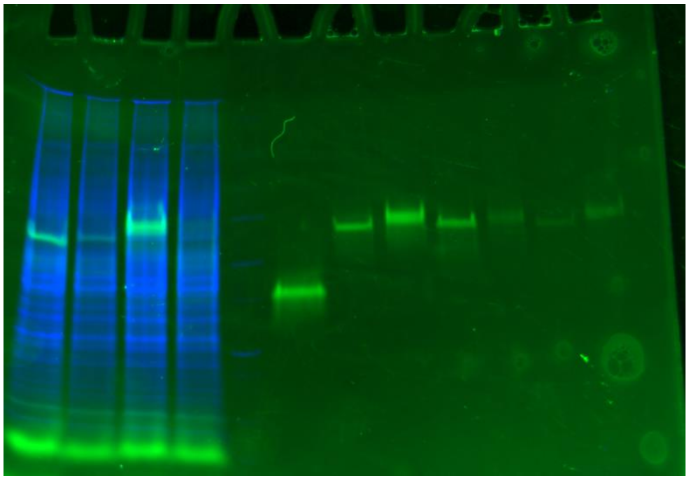

Extended Data Fig. 2c. Lanes 1-8 correspond to gel in paper.

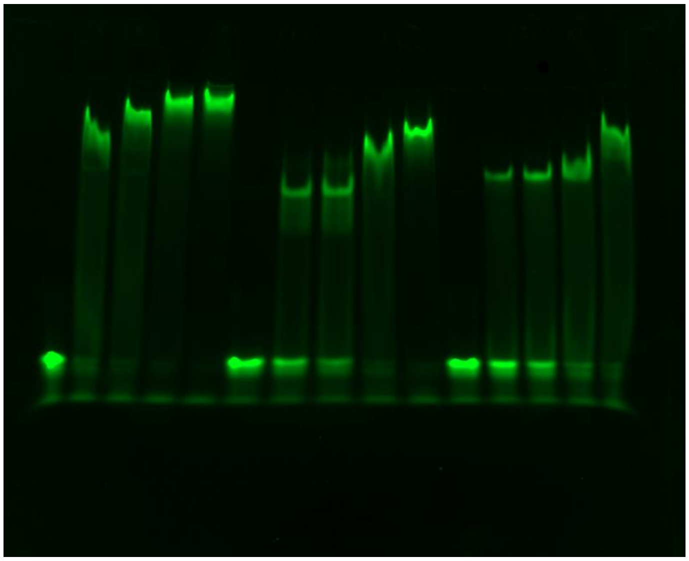

Extended Data Fig. 2d. Lanes 11-15 correspond to gel in paper.

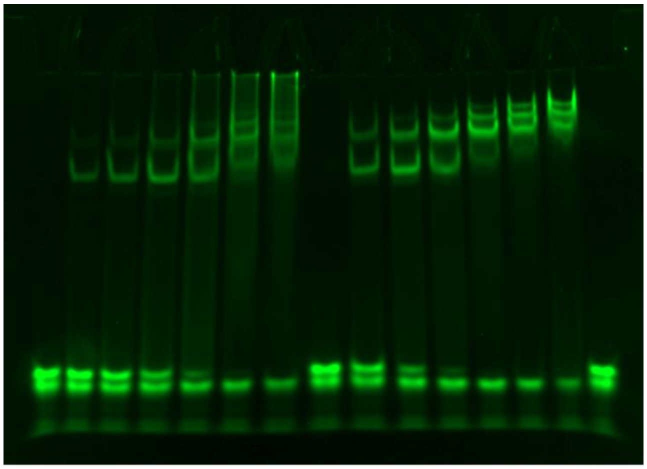

Extended Data Fig. 2e. Lanes 1-7 correspond to gel in paper.

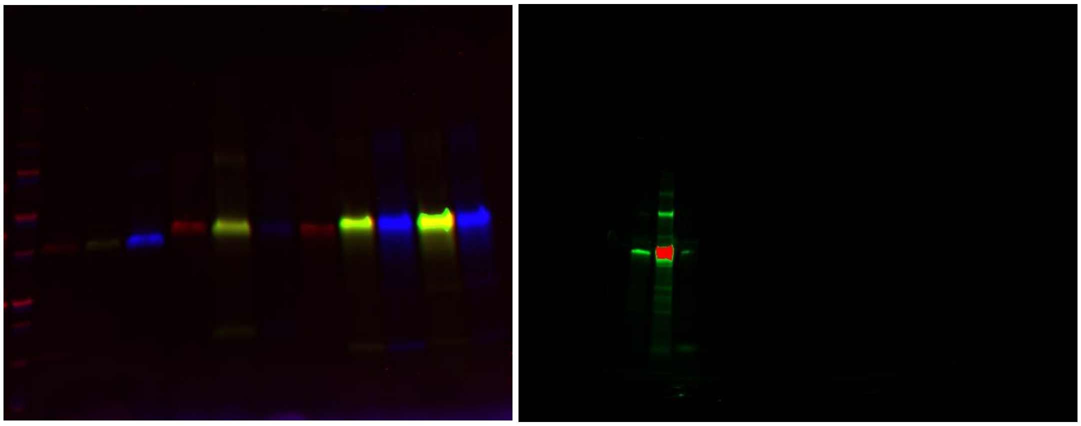

Extended Data Fig. 2f. Lanes 1-4,6 of left gel same correspond to lanes 1-4,5 in paper. Lane 1 of right gel correspond to lane 6 in paper.
