# Supplementary material for: NANOG prion-like assembly mediates DNA bridging to facilitate chromatin reorganization and activation of pluripotency
Source: Nat Cell Biol. 2022 Apr 28;24(5):737–47. doi: 10.1038/s41556-022-00896-x (PMC9106587; doi:10.1038/s41556-022-00896-x)
Supplement: Source Data Extended Data Fig. 5 — Unprocessed gels. [file 41556_2022_896_MOESM20_ESM.pdf]

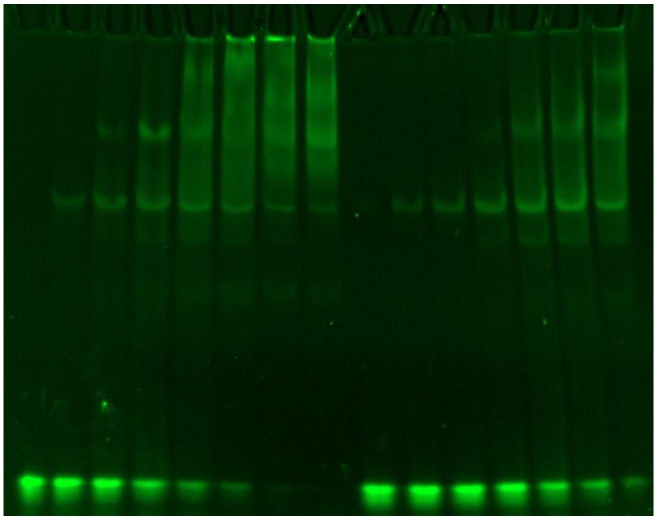

Extended Data Fig. 5a. Exact gel image in paper.

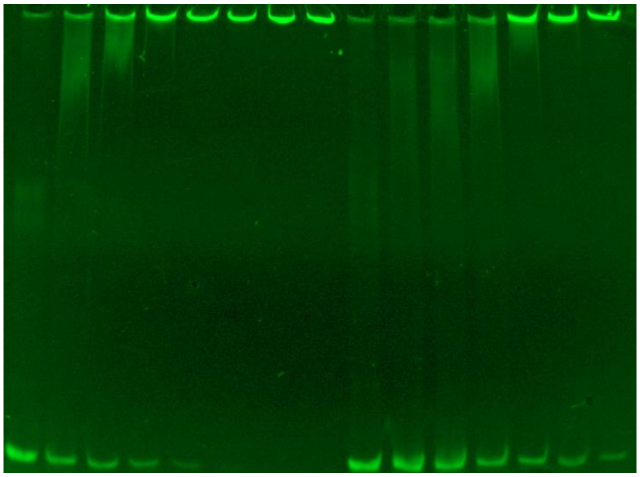

Extended Data Fig. 5b. Exact gel image in paper.

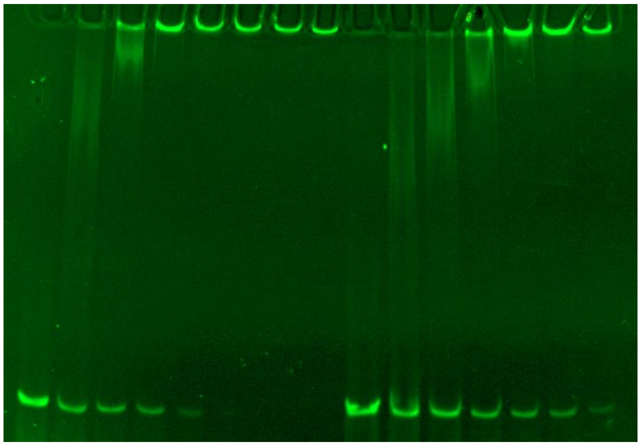

Extended Data Fig. 5c. Exact gel image in paper

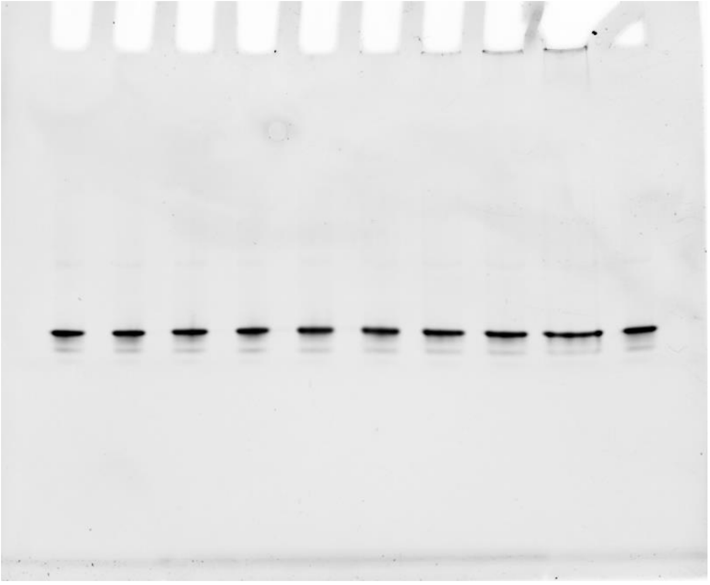

Extended Data Fig. 5d. Lanes 1-9 correspond to gel in paper.
